# Ulipristal acetate for Japanese women with symptomatic uterine fibroids: A double‐blind, randomized, phase II dose‐finding study

**DOI:** 10.1002/rmb2.12304

**Published:** 2019-10-30

**Authors:** Minoru Irahara, Yasuhiro Maejima, Nobuhiro Shinbo, Yuji Yamauchi, Hideki Mizunuma

**Affiliations:** ^1^ Tokushima University Tokushima Japan; ^2^ ASKA Pharmaceutical Co., Ltd Tokyo Japan; ^3^ Fukushima Medical University Fukushima Japan

**Keywords:** leiomyoma, menorrhagia, selective progesterone receptor modulator, ulipristal acetate, uterine fibroids

## Abstract

**Purpose:**

A multicenter, randomized, double‐blind, placebo‐controlled trial was conducted to evaluate the efficacy, safety, and appropriate dose of ulipristal acetate (UPA) in Japanese women with symptomatic uterine fibroids (UFs).

**Methods:**

A total of 121 premenopausal women with UFs were enrolled to receive either placebo, UPA‐2.5 mg, UPA‐5 mg, UPA‐10 mg, or leuprorelin acetate (LEU), a reference drug, for 12 weeks. The primary end point was the rate of patients having achieved amenorrhea for 35 days at Week 12.

**Results:**

The rates for amenorrhea were 4.5%, 60.0%, 72.7%, 88.0%, and 76.2% in the placebo, UPA‐2.5 mg, UPA‐5 mg, UPA‐10 mg, and LEU groups, respectively. The median times to amenorrhea were 20.0, 5.0, 5.0, and 23.0 days for treatment with UPA‐2.5 mg, UPA‐5 mg, UPA‐10 mg, and LEU, respectively. A significant dose‐response of UPA for the rate of amenorrhea was observed. The overall incidence rates of adverse events were 45.8% in the placebo group, 56.5%‐80.0% in the UPA groups, and 100.0% in the LEU group. There were no notable safety issues with UPA.

**Conclusions:**

Ulipristal acetate was effective and well tolerated in Japanese women with UFs. The recommended dose of UPA is considered to be 10 mg.

## Graphical abstract

This is the study design. In addition to a placebo and ulipristal acetate (UPA), leuprorelin (LEU) was used as a reference drug to evaluate the efficacy and safety of UPA. Patients were randomly assigned into five treatment groups. Patients were treated for 12 weeks and then followed up for 12 weeks. UPA and the placebo were administered in a double‐blind manner, and LEU, an injection drug, was administered in an open‐label manner. The median time to achieve amenorrhea for each group was 20.0, 5.0, 5.0, and 23.0 days for UPA‐2.5, UPA‐5, UPA‐10, and LEU, respectively.

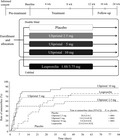

## 1 INTRODUCTION

Uterine fibroids (UFs) are common benign uterine smooth muscle tumors with a prevalence range of 4.5%‐68.6% depending on race and age.^1^ The cumulative incidence of UFs is reportedly 6.8% for women in their 40 seconds and 18.9% for those in their 50 seconds^2^; the number of patients treated is estimated to be around 550 000 in Japan.^3^ Menorrhagia, a burden for patients with UFs, limits daily activities, causes anemia, and associates with lower quality of life (QOL).^4^, ^5^ The radical treatment for UFs is total hysterectomy and conservative treatments such as myomectomy or pharmacotherapy are selected for patients who want to preserve the uterus or fertility.^6^ However, UFs often recur after myomectomy and the cumulative recurrence rates after myomectomy ranges from 11.7% to 84.4% depending on follow‐up time.^7^, ^8^ With regard to the pharmacotherapy, gonadotropin‐releasing hormone (GnRH) agonists or antagonist are the only drugs approved for UFs in Japan and provide effective therapeutic options. These drugs induce an artificial menopausal state that results in improving symptoms and reducing uterine and fibroid volume.^9^, ^10^ However, there are safety issues for both types of drugs concerning loss of bone mineral density due to low blood estrogen levels^10^, ^11^; therefore, the treatment duration is limited to up to 6 months in Japan. Moreover, patients treated with these drugs sometimes experience menopausal symptoms, such as hot flush.

Ulipristal acetate (UPA), a selective progesterone receptor (PR) modulator (SPRM), acts on PRs in myometrium, endometrium and hypothalamus–pituitary without major effects on estradiol (E2) levels.^12^, ^13^, ^14^, ^15^, ^16^, ^17^ UPA is considered to improve menorrhagia through induction of amenorrhea by inhibition of ovulation and direct action on the endometrium.^18^ Previous studies of UPA showed a bleeding control rate of 90%‐98% and a rate for amenorrhea of 73%‐89%.^19^, ^20^ Progesterone and PRs are involved in myometrial cellular proliferation and fibroid growth,^21^, ^22^ and SPRMs act antagonistically on the PRs of UFs.^23^, ^24^, ^25^ UPA reduced fibroid volume by 12%‐42% in phase III trials in Europe.^19^, ^20^ In addition, previous studies have shown that UPA achieves earlier amenorrhea, less menopausal symptoms, and less effect on bone metabolic makers compared with leuprorelin (LEU), a GnRH agonist.^20^ An SPRM is expected to solve the safety issues or concerns that may be associated with the hypoestrogenic effect of GnRH agonists and antagonists in the treatment of UFs. Ulipristal acetate was approved in Europe for UFs in 2012 and is currently used in 80 countries. In many countries, UPA is also indicated for long‐term intermittent treatment of UFs in premenopausal women who are not eligible for surgery.^21^ Herein, we report a phase II trial to evaluate the efficacy, safety, and appropriate dose of UPA in Japanese women with symptomatic UFs. This is the first randomized, controlled study of UPA for UFs in an Asian population.

## 2 MATERIALS AND METHOD

### 2.1 Design

**Figure 1:**
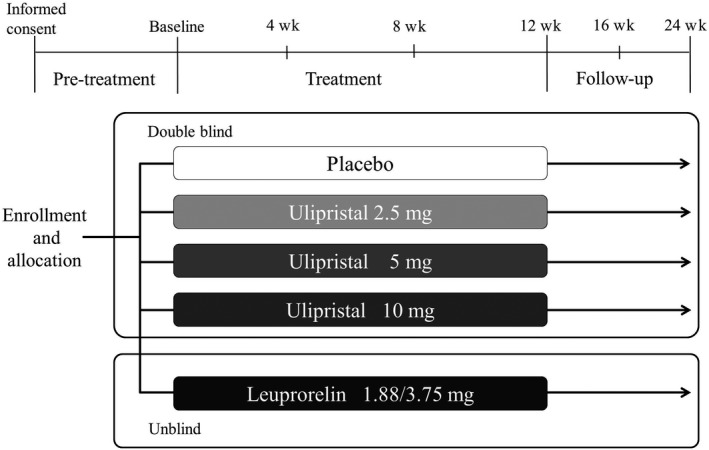
Study design

A multicenter, randomized, double‐blind, placebo‐controlled, parallel‐group trial was conducted in premenopausal women with UFs having menorrhagia symptoms at 38 sites in Japan. In addition to a placebo and UPA, LEU was used as a reference drug to evaluate the efficacy and safety of UPA. Under a central registration, eligible patients were randomly assigned into five treatment groups—placebo, UPA 2.5 mg (UPA‐2.5), UPA 5 mg (UPA‐5), UPA 10 mg (UPA‐10), and LEU—using an allocating factor of the presence or absence of iron therapy (Figure 1). Patients were treated for 12 weeks and then followed up for 12 weeks. Ulipristal acetate and the placebo were administered in a double‐blind manner, and LEU, an injection drug, was administered in an open‐label manner. The examination schedule is shown in Table S1.

The following committees were established: the Centralized Gynecological Assessment Committee for the evaluation of amenorrhea, the Centralized Image Assessment Committee for the evaluation of the MRI images, and the Centralized Histopathological Assessment Committee for the pathological diagnosis.

### 2.2 Intervention

In the placebo and UPA groups, study drugs were orally administered once a day for 12 weeks. In the LEU group, 1.88 or 3.75 mg of LEU was subcutaneously administered once every 4 weeks. The dose of LEU for each patient was determined by the investigator based on body weight and fibroid size. The dosing started within 7 days of menstruation in the placebo and UPA groups and 5 days in the LEU group. The following medications were prohibited during the study: GnRH analogues, systemic corticosteroids, progestin, oral contraceptives, anticoagulants (acetylsalicylic acid, mefenamic acid, and coumarin), antifibrinolytic agents (tranexamic acid), and strong CYP3A4 inhibitors or inducers. Iron therapy was prohibited during the study for patients who had not received it at the time of giving informed consent, except when the investigator judged its necessity. For those patients who had received iron therapy for 4 weeks prior to giving informed consent, it was continued without a dose modification during the study.

### 2.3 End points

The primary end point was defined as the rate of patients having achieved amenorrhea for 35 consecutive days (from Day 50 to Day 84) at Week 12. The bleeding status was evaluated according to four grades—none, spotting, bleeding, and heavy bleeding—and patients recorded daily bleeding status using electronic devices. Amenorrhea was defined as the absence of heavy bleeding or bleeding during the assessment period. Amenorrhea was also assessed from bleeding status, basal body temperature, and hormone levels by the Centralized Gynecological Assessment committee as an exploratory end point.

Secondary end points were as follows: (a) time to achieve amenorrhea, (b) the proportion of patients achieving amenorrhea for 56 consecutive days (from Day 29 to Day 84) at Week 12, (c) the rate of patients with controlled uterine bleeding from Day 29 to Day 84 (bleeding 8 days or less and no heavy bleeding), (d) time to recovery of menstruation, (e) rate of change in total volume of three largest fibroids and uterine volume, (f) rate of change in Hb level, and (g) rate of change in anemia‐related items, visual analogue scale (VAS), and QOL status using health survey SF‐36v2^®^.^26^, ^27^, ^28^

### 2.4 Safety end points

The safety end points were as follows: (a) adverse events (AEs) coded using the Medical Dictionary for Regulatory Activities/Japanese (MedDRA/J) Ver. 19.1 and tabulated by System Organ Class and Preferred Terms, (b) E2 and progesterone levels, (c) change in endometrial thickness, (d) histological diagnosis of endometrium, (e) laboratory tests (hematology and biochemistry), (f) endocrine test (adrenocorticotropic hormone (ACTH), thyrotropin‐releasing hormone (TSH), follicle‐stimulating hormone (FSH), luteinizing hormone (LH), and prolactin), and (g) bone turnover markers (blood type I procollagen N terminal propeptide [PINP], blood bone alkaline phosphatase [BAP], urine deoxypiridinoline, and urine type I collagen C terminal telopeptide [CTX]).

### 2.5 Patient eligibility

Women with UFs who met the following inclusion criteria and did not exhibit any of the exclusion criteria were eligible for this study. Inclusion criteria were as follows: (a) Japanese premenopausal women aged 20‐50 years old, (b) menstrual cycle ranging from 22 to 35 days, (c) menorrhagia with heavy bleeding for more than 1 day within 8 days from the start of menstruation, (d) hemoglobin level between ≥6.0 g/dL and <11.5 g/dL, (e) patients with one or more myoma ≥3 cm and no myoma >12 cm by pelvic MRI diagnosis, and (f) patients scheduled for surgery (hysterectomy or myomectomy) after the treatment period. Exclusion criteria were as follows: (a) a history of uterine surgery affecting the evaluation of this study (excluding cesarean section and cervical conization) or having undergone intrauterine curettage, arterial embolization, or microwave endometrial ablation, (b) cervical cancer, uterine cancer, ovarian cancer, breast cancer, endometrial hyperplasia, or a history or suspected of having these diseases, (c) calcified myoma nucleus, (d) hemoglobinopathy or severe coagulation disorder, (e) pregnant or breast‐feeding, positive pregnancy test at screening, or with the hope of a pregnancy during the study, (f) liver dysfunction, and (g) the possibility of the use of any of the prohibited drugs during the study.

### 2.6 Statistics

#### 2.6.1 Sample size

Based on the results of a previous study,^18^ the rate of amenorrhea was estimated to be 18.2%, 63.3%, 80.0%, and 0% for UAP‐2.5, UPA‐5, UPA‐10, and placebo, respectively. Under conditions of a power of >85% and a significance level of dose‐response of <0.025 (one‐sided), the number of patients required for the superiority of UPA‐5 to placebo to be <0.05 was estimated to be 11 cases per group. Taking into consideration withdrawal from the study, the number of samples required to evaluate the efficacy and safety of UPA was set to be 25 cases per group.

#### 2.6.2 Analysis population

Efficacy was evaluated using the full analysis set (FAS). The FAS was defined as the population excluding the following patients from the enrolled population: (a) patients who did not take study drugs, (b) ineligible patients, and (c) patients having no data for efficacy. Safety was assessed using the safety analysis set (SAF). The SAF was defined as the patient population who took at least one dose of study drug.

#### 2.6.3 Procedure

Descriptive statistics were expressed as n (%), mean ± SD, % [95% CI], and median [95% CI]. The dose‐response of UPA was evaluated using the Cochran‐Armitage test. Superiority of each UPA group to the placebo group was evaluated using Fisher's exact test under closed testing procedure. The time to achieve amenorrhea and the time to recovery of menstruation were estimated using the Kaplan‐Meier method, and comparisons between groups were performed using the log‐rank test. To evaluate the rate of change in total fibroid volume, uterine volume, and hemoglobin level, repeated measure analysis of variance was performed using group, time, and time and group interaction as explanatory variables. As LEU was reference drug, statistical test between UPA groups and LEU group were not performed. Alpha (α) was set to be 0.05 (both‐sided). For the Cochran‐Armitage test, α was set to 0.025 (one‐sided). Statistics were determined using SAS Release 9.3 (SAS Institute Inc).

## 3 RESULTS

### 3.1 Patient characteristics

**Figure 2:**
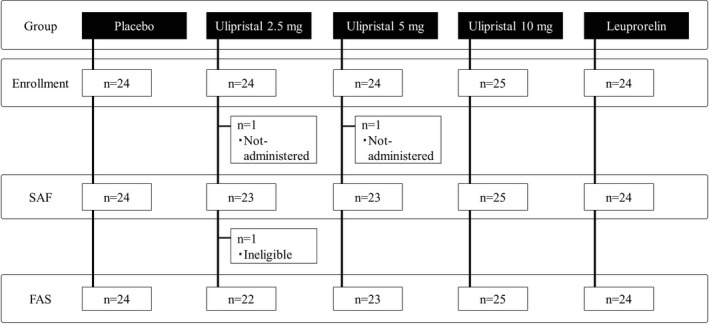
Patient disposition. FAS, full analysis set; SAF, safety analysis set

Patient disposition is shown in Figure 2. A total of 121 patients were enrolled in this study. Two patients who did not receive any study drug were excluded from the SAF, and one patient who was ineligible for the study was excluded from the FAS. The number of patients included in the SAF/FAS for each treatment group was 24/24, 23/22, 23/23, 25/25, and 24/24 for the placebo, UPA‐2.5, UPA‐5, UPA‐10, and LEU groups, respectively. No differences other than social/role scores in QOL were found in patient characteristics between each UPA group and the placebo group (Table 1).

**Table 1:** Characteristics of the full analysis set participants

	Placebo	Ulipristal			*P*	Leuprorelin
		2.5 mg	5 mg	10 mg		
FAS (n)	24	22	23	25		24
Age (y.o.), mean ± SD	42.6 ± 4.0	40.4 ± 5.5	40.7 ± 5.9	41.1 ± 5.2	.4615	43.2 ± 4.2
BMI (kg/m^2^), mean ± SD	20.80 ± 2.96	22.68 ± 3.11	22.63 ± 3.50	22.61 ± 4.54	.2072	22.53 ± 3.86
Menstrual cycle (d), mean ± SD	28.4 ± 1.6	27.7 ± 1.4	28.7 ± 2.2	28.2 ± 2.4	.4388	28.2 ± 2.3
Total volume of the three largest uterine fibroid (cm^3^)	154.2 ± 153.9	207.3 ± 200.1	220.3 ± 187.3	162.7 ± 145.6	.4755	214.8 ± 200.7
Uterine volume (cm^3^)	378.7 ± 251.5	406.1 ± 329.7	459.0 ± 343.2	443.5 ± 307.3	.8059	520.0 ± 426.2
Iron therapy, n (%)						
No	18 (75.0%)	17 (77.3%)	18 (78.3%)	19 (76.0%)	.9941	18 (75.0%)
Yes	6 (25.0%)	5 (22.7%)	5 (21.7%)	6 (24.0%)		6 (25.0%)
Hemoglobin (g/dL)	9.82 ± 1.45	9.50 ± 1.62	9.93 ± 1.11	9.73 ± 1.76	.9254	9.42 ± 2.26
Without iron therapy	10.43 ± 0.51	10.37 ± 0.88	10.17 ± 0.72	9.75 ± 0.98	.1554	9.50 ± 1.22
With iron therapy	9.73 ± 1.53	9.09 ± 1.75	9.82 ± 1.25	9.73 ± 1.97	.7606	9.36 ± 2.82
QOL						
Physical	48.13 ± 10.21	46.62 ± 9.33	46.77 ± 9.05	46.18 ± 9.46	.5026	49.22 ± 6.14
Mental	46.48 ± 8.02	48.33 ± 7.43	48.89 ± 8.54	48.86 ± 9.45	.3147	47.65 ± 8.3
Role/Social	41.96 ± 11.24	47.43 ± 9.77	48.07 ± 13.41	50.32 ± 8.89	.0103	48.09 ± 9.65

Abbreviations: FAS, full analysis set; QOL, quality of life.

### 3.2 Efficacy

**Figure 3:**
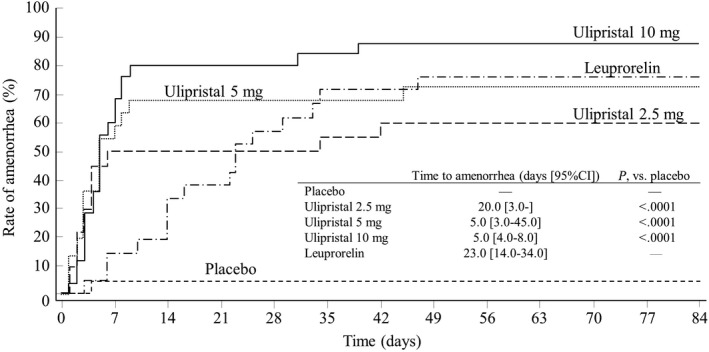
Time to amenorrhea

The rate of patients with 35 consecutive days of amenorrhea at Week 12 for each group were 4.5%, 60.0%, 72.7%, 88.0%, and 76.2% for the placebo, UPA‐2.5, UPA‐5, UPA‐10, and LEU groups, respectively, and were significantly higher in all of the UPA groups compared with the placebo group (Table 2). A significant dose‐response of UPA for the rate of 35 days of amenorrhea at Week 12 was observed. Similar results were observed for amenorrhea based on the Centralized Gynecological Assessment, an exploratory end point (data not shown). The median time to achieve amenorrhea for each group was 20.0, 5.0, 5.0, and 23.0 days for UPA‐2.5, UPA‐5, UPA‐10, and LEU, respectively (Figure 3). The rates for 56 consecutive days of amenorrhea at Week 12 and the rates of controlled uterine bleeding from Day 29 to Day 84 were significantly higher in all UPA groups compared with the placebo group, and significant dose‐responses of UPA were observed (Table 2). The median time to menstrual recovery was 21.0, 24.0, 29.0, and 60.0 days for UPA‐2.5, UPA‐5, UPA‐10, and LEU, respectively. A significant dose‐response of UPA for the reduction rates of the total volume of the three largest UFs was observed. There were no significant dose‐responses of UPA for the rate of change in uterine volume, the rate of change in hemoglobin level (Table 2), hematocrit, mean corpuscular volume, ferritin, total iron‐binding capacity (Table S2), change in the VAS score for pain (Table S3) or any of the three component summaries for QOL (Table S4).

**Table 2:** Efficacy end points for the full analysis set

	Placebo	Ulipristal			Leuprorelin
		2.5 mg	5 mg	10 mg	
FAS, n	24	22	23	25	24
Rate of amenorrhea at 12 wk (35 d), % [95% CI] (n)	4.5 [0.1‐22.8] (22)	60 [36.1‐80.9] (20)	72.7 [49.8‐89.3] (22)	88.0 [68.8‐97.5] (25)	76.2 [52.8‐91.8] (21)
*P* value vs placebo	—	.0001	<.0001	<.0001	—
*P* value for trend*	<.0001				—
Rate of amenorrhea at 12 wk (56 d), % [95% CI] (n)	4.5 [0.1‐22.8] (22)	50.0 [27.2‐72.8] (20)	68.2 [45.1‐86.1] (22)	80.0 [59.3‐93.2] (25)	61.9 [38.4‐81.9] (21)
*P* value vs placebo	—	.0012	<.0001	<.0001	—
*P* value for trend*	<.0001				—
Rate of patients with controlled uterine bleeding from 29 to 84 d, % [95% CI] (n)	5.0 [0.1‐24.9] (20)	70.0 [45.7‐88.1] (20)	81.0 [58.1‐94.6] (21)	96.0 [79.6‐99.9] (25)	85.0 [62.1‐96.8] (20)
*P* value vs placebo	—	<.0001	<.000	<.0001	—
*P* value for trend*	<.0001				—
Time from the end of administration to recovery of menstruation (d), median [95% CI] (n)	75 (1)	21.0 [8.0‐30.0] (12)	24.0 [20.0‐31.0] (16)	29.0 [21.0‐47.0] (22)	60.0 [46.0‐75.0] (16)
*P* value vs placebo	—	.0796	.067	.599	—
*P* value for trend*	.0537				—
Rate of change in total volume of the three largest uterine fibroids (%), mean ± SD (n)					
12 wk	−0.33 ± 30.90 (20)	−6.64 ± 20.61 (22)	−13.87 ± 36.46 (21)	−25.06 ± 40.00 (25)	−34.40 ± 24.80 (23)
16 wk	−4.25 ± 22.31 (13)	−10.18 ± 30.46 (19)	−11.88 ± 29.27 (19)	−31.99 ± 24.50 (15)	−29.58 ± 26.65 (15)
24 wk	−1.97 ± 29.44 (5)	12.25 ± 27.88 (11)	−22.49 ± 28.39 (11)	−39.76 ± 39.96 (8)	−0.81 ± 36.64 (6)
*P* value for trend*	<.0001				—
Rate of change in uterine volume (%), mean ± SD (n)					
12 wk	3.38 ± 26.16 (20)	−0.21 ± 32.55 (22)	−3.35 ± 36.51 (21)	−22.18 ± 19.80 (25)	−36.15 ± 13.96 (23)
16 wk	1.21 ± 20.67 (13)	−0.29 ± 27.84 (19)	−0.14 ± 37.04 (19)	−12.64 ± 24.47 (15)	−34.45 ± 20.53 (15)
24 wk	−3.34 ± 26.54 (5)	13.76 ± 32.06 (11)	24.20 ± 87.20 (10)	−4.74 ± 29.96 (8)	−12.38 ± 27.87 (6)
*P* value for trend*	.1985				—
Rate of change in hemoglobin level (%), mean ± SD (n)					
Total (n = 118)					
12 wk	13.43 ± 27.85 (20)	16.76 ± 23.71 (22)	17.97 ± 12.12 (21)	22.36 ± 22.54 (25)	29.07 ± 30.55 (23)
*P* value for trend*	>.025				—
Patients with iron therapy (n = 85)					
12 wk	15.03 ± 28.98 (18)	21.00 ± 26.70 (15)	22.16 ± 12.75 (14)	24.58 ± 24.62 (19)	41.10 ± 36.10 (13)
*P* value for trend*	>.025				—
Patients without iron therapy (n = 33)					
12 wk	−0.95 ± 0.06 (2)	7.65 ± 12.77 (7)	9.58 ± 3.79 (7)	15.33 ± 13.37 (6)	13.42 ± 7.85 (10)
*P* value for trend*	>.025				—

Abbreviation: FAS, full analysis set.

* *P* for trend between placebo and UPA groups.

### 3.3 Safety

The incidence rates of AEs were 45.8%, 56.5%, 73.9%, 80.0%, and 100.0% for the placebo, UPA‐2.5, UPA‐5, UPA‐10, and LEU treatment groups, respectively (Table 3). Drug‐related AEs found in more than 5% of any group were constipation, edema, endometrial hyperplasia, menopausal symptoms, menorrhagia, metrorrhagia, and hot flush. Among these AEs, the incidence rates of menopausal symptoms, menorrhagia, metrorrhagia, and hot flush were lower in each of the UPA groups compared with the LEU group. The most common AE in the UPA groups was nasopharyngitis (21.7%, UPA‐2.5), followed by constipation (12.0%, UPA‐10), endometrial hyperplasia (8.7%, UPA‐2.5), and hot flush (8.7%, UPA‐5). The histopathology of all endometrial hyperplasia found in the UPA groups was judged as benign endometrium by the Centralized Histopathological Assessment Committee. Serious AEs were reported in two patients in the placebo group (ileus paralytic and breast cancer), one patient in the UPA‐5 group (hemorrhagic anemia), and two patients in the LEU group (peritonitis and gastroduodenal ulcer). No serious AEs were reported in the UPA‐2.5 or UPA‐10 groups. Hemorrhagic anemia, a serious AE, observed in the UPA‐5 group was considered to be due to the myomectomy after the treatment period; a causal relationship with the UPA was ruled out.

**Table 3:** Adverse events in the safety analysis set

	Placebo	Ulipristal			Leuprorelin
		2.5 mg	5 mg	10 mg	
SAF (n)	24	23	23	25	24
Total, n (%)	11 (45.8%)	13 (56.5%)	17 (73.9%)	20 (80.0%)	24 (100.0%)
Drug related, n (%)	6 (25%)	8 (34.8%)	12 (52.2%)	9 (36.0%)	20 (83.3%)
Drug‐related AEs ≥5% in any group, n (%)					
Constipation	0	1 (4.3%)	1 (4.3%)	2 (8.0%)	0
edema	0	0	2 (8.7%)	1 (4.0%)	0
Endometrial hyperplasia†	0	2 (8.7%)	1 (4.3%)	1 (4.0%)	0
Menopausal symptoms	0	0	0	0	3 (12.5%)
Menorrhagia	0	0	0	0	3 (12.5%)
Metrorrhagia	0	1 (4.3%)	1 (4.3%)	2 (8.0%)	10 (41.7%)
Hot flush	0	0	2 (8.7%)	0	7 (29.2%)

Abbreviations: AE, adverse event; SAF, safety analysis set.

^†^ The histopathology of four cases of endometrial hyperplasia found in the UPA groups was judged as benign endometrium by the Centralized Histopathological Assessment Committee.

Changes in E2, progesterone levels and endometrial thickness are shown in Table 4. Since the study drugs were started during menstruation, the E2 and progesterone levels showed a decrease at the baseline compared to pre‐treatment in all groups. The E2 levels returned to the pre‐treatment levels at Week 8 in the placebo and UPA groups while it decreased through Week 12 in the LEU group. During the treatment period, most progesterone levels in the UPA groups were lower than those in the placebo group; in particular, those in the UPA‐10 group were maintained at the baseline level as with the LEU group. The FSH levels showed a decrease from Week 4 through Week 12 in all UPA groups and the LEU group and LH levels decreased during the treatment period in the LEU group (Table S5). There were no changes in other endocrine tests (ACTH, TSH, and prolactin). The endometrial thickness did not show apparent changes from the pre‐treatment period to Week 24 in the placebo and the UPA groups. In the LEU group, endometrial thickness became thinner at Week 12 and then recovered by Week 24 to the pre‐treatment level.

**Table 4:** Changes in estradiol and progesterone levels and endometrium thickness in the safety analysis set

	Placebo	Ulipristal			Leuprorelin
		2.5 mg	5 mg	10 mg	
SAF (n)	24	23	23	25	24
Estradiol (pg/mL), mean ± SD (n)					
Pre‐treatment	165.3 ± 159.0 (24)	111.9 ± 86.8 (23)	135.7 ± 130.1 (23)	110.4 ± 74.9 (25)	141.5 ± 123.5 (24)
Base line	51.3 ± 41.2 (24)	55.0 ± 47.1 (23)	61.3 ± 69.7 (23)	41.3 ± 26.8 (25)	48.5 ± 38.4 (24)
4 wk	87.1 ± 61.2 (24)	73.3 ± 59.2 (23)	100.3 ± 105.9 (23)	116.7 ± 73.4 (25)	8.0 ± 5.6 (24)
8 wk	133.5 ± 113.3 (22)	143.5 ± 130.2 (23)	162.5 ± 150.7 (23)	93.1 ± 93.2 (25)	11.0 ± 12.7 (24)
12 wk	137.7 ± 140.1 (20)	113.4 ± 92.5 (23)	107.7 ± 72.9 (21)	102.6 ± 99.1 (25)	12.0 ± 10.2 (23)
16 wk	133.3 ± 92.8 (19)	94.3 ± 80.7 (21)	125.5 ± 86.0 (21)	137.0 ± 150.9 (25)	112.0 ± 221.6 (22)
24 wk	130.8 ± 104.7 (18)	143.6 ± 100.8 (21)	108.1 ± 94.7 (21)	107.4 ± 77.0 (25)	117.5 ± 135.0 (22)
Progesterone (ng/mL), mean ± SD (n)					
Pre‐treatment	5.383 ± 6.277 (24)	3.717 ± 5.809 (23)	2.639 ± 4.154 (23)	4.192 ± 6.414 (25)	3.242 ± 4.648 (24)
Base line	0.483 ± 0.617 (24)	0.230 ± 0.118 (23)	0.278 ± 0.217 (23)	0.320 ± 0.196 (25)	0.296 ± 0.266 (24)
4 wk	1.821 ± 3.344 (24)	2.039 ± 3.669 (23)	1.257 ± 3.502 (23)	0.734 ± 1.823 (25)	0.133 ± 0.075 (24)
8 wk	4.061 ± 6.164 (22)	2.915 ± 4.678 (23)	0.811 ± 2.610 (23)	0.780 ± 2.269 (25)	0.146 ± 0.059 (24)
12 wk	4.410 ± 5.766 (20)	1.774 ± 3.409 (23)	1.524 ± 3.298 (21)	0.204 ± 0.110 (25)	0.152 ± 0.067 (23)
16 wk	3.742 ± 5.030 (19)	3.824 ± 5.963 (21)	1.529 ± 2.674 (21)	2.888 ± 4.978 (25)	0.925 ± 3.529 (22)
24 wk	3.564 ± 4.462 (18)	4.452 ± 7.188 (21)	4.919 ± 5.984 (21)	2.292 ± 3.982 (25)	5.455 ± 8.343 (22)
Endometrial thickness (mm), mean ± SD (n)					
Pre‐treatment	8.38 ± 4.86 (24)	8.00 ± 4.18 (23)	9.73 ± 4.21 (23)	9.71 ± 4.15 (25)	10.01 ± 4.56 (24)
12 wk	8.54 ± 4.80 (20)	7.96 ± 5.53 (23)	9.51 ± 5.14 (21)	9.52 ± 6.18 (25)	4.03 ± 2.14 (23)
16 wk	7.95 ± 2.85 (15)	8.50 ± 4.41 (20)	9.53 ± 6.36 (21)	10.98 ± 7.81 (22)	7.01 ± 4.77 (18)
24 wk	8.83 ± 3.40 (8)	9.28 ± 4.73 (16)	10.31 ± 4.81 (17)	10.66 ± 5.94 (18)	9.59 ± 4.55 (10)

Abbreviation: SAF: safety analysis set.

Evaluation of the endometrial histopathology by the Centralized Histopathological Assessment Committee showed that the percentage of patients diagnosed with non‐physiological changes in endometrial histopathology transiently increased at Week 12 in each UPA group and then returned to the pre‐treatment levels.

The bone turnover markers (PINP, BAP, deoxypiridinoline, and CTX) are shown in Table S6. No notable changes were observed in the laboratory test values including the hematology, biochemistry, and bone turnover markers in the UPA groups. An upward trend was observed in the LEU group for Na, Ca, alkaline phosphatase (data not shown), and urine CTX.

## 4 DISCUSSION

In the present study, the efficacy and safety of a dose range of 2.5‐10 mg of UPA for 12 weeks was evaluated in Japanese women with symptomatic UFs. The rate of achieving amenorrhea and controlled uterine bleeding were significantly higher in all UPA groups compared with the placebo group. Significant dose‐response of UPA was observed in the rate of amenorrhea, the time to amenorrhea, the rate of patients with controlled uterine bleeding, and the reduction rate of total volume of the three largest UFs. A dose range of 2.5‐10 mg of UPA appears to be safe and well tolerated.

The rates of patients with amenorrhea for 35 days at Week 12 were 4.5%, 60.0%, 72.7%, 88.0%, and 76.2% for the placebo, UPA‐2.5, UPA‐5, UPA‐10, and LEU groups, respectively, and the median time of days to amenorrhea was 20.0, 5.0, 5.0, and 23.0 for the UPA‐2.5, UPA‐5, UPA‐10, and LEU groups, respectively. These results were comparable to the previous studies of UPA conducted in Europe.^19^, ^20^ UPA is considered to improve menorrhagia through induction of amenorrhea by inhibition of ovulation and direct action on the endometrium.^18^, ^29^ The low progesterone levels observed in the UPA groups in the present study may reflect the absence of luteinization along with suppression of ovulation. During the treatment period, the progesterone levels in UPA‐10 group were maintained at the baseline (menstrual phase) level, while those in UPA‐2.5 and 5 groups were slightly higher than UPA‐10 group. These findings may reflect dose response of amenorrhea rate in the UPA groups. The E2 levels in the UPA groups were maintained at follicular phase levels during the treatment period. Since UPA did not cause flare‐up, it was suggested that the time to amenorrhea is short in the UPA groups. We believe that early achievement of amenorrhea by UPA will be a therapeutic advantage over GnRH agonists. There were no significant differences in the rates of change in Hb between the UPA groups and the placebo group, and the reason may be that patients without iron therapy were relatively few in the placebo group. However, the UPA‐10 group showed an increase in Hb level similar to that of the LEU group, indicating an improvement in anemia.

The mean reduction rates of total volume of the three largest UFs were −13.87% for UPA‐5, −25.06% for UPA‐10, and −34.40% for LEU. These reduction rates were less than those reported in the PEARL II, a phase III trial of UPA vs leuprolide, and similar to those reported in the PEARL I, a phase III trial of UPA vs placebo.^19^, ^20^

The overall incidence rates of AEs were 56.5%, 73.9%, 80.0%, and 100.0% for the UPA‐2.5, UPA‐5, UPA‐10, and LEU groups, respectively. The most common AE in the UPA groups was nasopharyngitis (21.7%, UPA‐2.5), followed by constipation (12.0%, UPA‐10), endometrial hyperplasia (8.7%, UPA‐2.5), and hot flush (8.7%, UPA‐5). All cases of endometrial hyperplasia found in the UPA groups were judged by the Centralized Histopathological Assessment Committee to be benign endometrium. In the LEU group, the most common AE was metrorrhagia (41.7%), followed by hot flush (29.2%), nasopharyngitis (20.8%), headache (12.5%), menopause‐related conditions (12.5%), and hypermenorrhea (12.5%). The AEs in the LEU group were considered to be mainly associated with low estrogen levels or flare‐up.

Endometrial modifications by SPRM are known as progesterone receptor modulator‐associated endometrial changes (PAEC).^30^, ^31^, ^32^ PAEC, considered to be non‐physiological changes, shows apoptosis, low mitotic activity in the glands and stroma, cystic glandular dilatation, absence of stromal breakdown and glandular crowding, and endometrial thickening is rarely observed.^30^, ^33^ As a mechanism of this morphological change, the modulation of E2 and progesterone by SPRM in endometrial stromal cells has been suggested.^34^ However, PAEC is reportedly benign and reversible from the interruption of SPRM administration or detachment of the endometrium during resumed menstruation.^33^, ^35^ Moreover, it has been reported that the endometrium recovers to its pre‐administration state even after repeated 3‐month administrations of UPA.^36^, ^37^, ^38^ As expected from these findings, the proportion of patients with non‐physiological endometrial pathology temporarily increased and then recovered. Furthermore, endometrial thickness in the UPA groups did not show apparent changes from pre‐treatment period and was similar to that in the placebo group. Therefore, we concluded that there were no noteworthy safety concerns.

A recent post‐marketing report on UPA showed rare cases of serious liver injury and hepatic failure and a suspected relationship to UPA; thus, assessment of the benefits and safety of UPA is considered to be necessary.^39^ In our study, we observed one case of hepatic function disorder in the UPA‐2.5 group, one case each of hepatic function disorder and increase in liver function test value in the UPA‐5 group, and one case of γ‐glutamyl transpeptidase increase in the UPA‐10 group. All of these AEs were mild and alanine aminotransferase and/or aspartate transaminase levels were lower than three times the upper limit of normal. No severe AEs related to the liver were observed.

This study has the following limitations. This was a dose‐finding study and the number of patients was limited. The study was conducted only with patients scheduled for surgery, and the follow‐up data for efficacy were limited by surgery performed during the follow‐up period. This study consisted of a 12‐week treatment phase and a 12‐week follow‐up phase, and thus, long‐term efficacy and safety in Japanese women were not clarified.

In the present study, UPA was effective and well tolerated and the rate of amenorrhea was significantly higher for all UPA groups compared with the placebo and a significant dose‐response was observed. There were no notable safety issues with UPA and no differences in the safety evaluations between the UPA groups. Therefore, the recommended dose of UPA for Japanese women with UFs was considered to be 10 mg. Ulipristal acetate does not reduce the estrogen level, unlike GnRH agonists, therefore, AEs due to hypoestrogen are unlikely to occur. Ulipristal acetate will be a new treatment option for Japanese women with uterine fibroids. Further confirmatory study to evaluate efficacy and safety of UPA is warranted. Currently, two UPA phase 3 studies are ongoing: one is a comparative study with LEU as a control, and the other is a long‐term study.

## CONFLICT OF INTEREST

Minoru Irahara, Yasuhiro Maejima, Yuji Yamauchi, Nobuhiro Shinbo and Hideki Mizunuma declare that he has no conflicts of interest.

## ETHICAL APPROVAL

The study protocol was approved by the ethics committee at each institution.

## HUMAN RIGHTS STATEMENTS AND INFORMED CONSENT

This study was carried out in compliance with the articles of the revised Declaration of Helsinki October 2013 and according to the Good Clinical Practices established by the Ministry of Health, Labor, and Welfare in Japan. The investigator provided an explanation sufficient to ensure that each patient clearly understood the study before obtaining written informed consent.

## CLINICAL TRIAL REGISTRY

The trial registration number is JapicCTI‐142718.

## STUDY ORGANIZATION

Study organization, sites and investigators are shown in Table S7.
