# Supplementary material for: Ulipristal acetate for Japanese women with symptomatic uterine fibroids: A double‐blind, randomized, phase II dose‐finding study
Source: Reprod Med Biol. 2019 Oct 30;19(1):65–74. doi: 10.1002/rmb2.12304 (PMC6955589; doi:10.1002/rmb2.12304)
Supplement: Supplementary file 1 [file RMB2-19-65-s001.docx]

Supporting Table 1. Examination schedule

|  | | Pre-treatment period | Treatment period | | | | Follow-up period | |
| --- | --- | --- | --- | --- | --- | --- | --- | --- |
|  |  |  | Base line | 4 weeks | 8 weeks | 12 weeks | 16 weeks | 24 weeks |
| Informed consent | | X |  |  |  |  |  |  |
| Registration | | X |  |  |  |  |  |  |
| Patient characteristics | | X |  |  |  |  |  |  |
| Study drug administration | |  | X | X | X | X† |  |  |
| Physical examination | | X | X | X | X | X | X | X |
| Electrocardiogram | | X |  |  |  | X | X |  |
| Gynecological examination | |  |  |  |  |  |  |  |
|  | Pelvic MRI scan | X |  |  |  | X | X | X |
|  | Breast examination | X |  |  |  | X |  | X |
|  | Cervical cytology | X |  |  |  | X |  |  |
|  | Endometrium histopathology | X |  |  |  | X |  | X |
| Pregnancy testing | | X | X |  |  | X |  |  |
| Laboratory test | |  |  |  |  |  |  |  |
|  | Hematology | X | X | X | X | X | X | X |
|  | Biochemical examination | X | X | X | X | X | X | X |
| Endocrine examination | | X | X | X | X | X | X | X |
| Bone metabolism marker | |  | X |  | X | X |  |  |
| Menstrual investigation | | X | X | X | X | X | X | X |
| Patient diary | |  |  |  |  |  |  |  |
|  | Bleeding investigation | X | X | X | X | X | X | X |
|  | Basal body temperature measuring | X | X | X | X | X | X | X |
|  | Compliance check | X | X | X | X | X | X | X |
|  | Pain assessment |  | X | X | X | X |  | X |
|  | QOL evaluation |  | X | X |  | X |  | X |
| Adverse event | | X | X | X | X | X | X | X |

†Excluding the leuprorelin group
