# Supplementary material for: Ulipristal acetate for Japanese women with symptomatic uterine fibroids: A double‐blind, randomized, phase II dose‐finding study
Source: Reprod Med Biol. 2019 Oct 30;19(1):65–74. doi: 10.1002/rmb2.12304 (PMC6955589; doi:10.1002/rmb2.12304)
Supplement: Supplementary file 2 [file RMB2-19-65-s002.docx]

Supporting Table 2. Rate of change in hematologic tests in the full analysis set

|  | | Placebo | Ulipristal | | | Leuprorelin |
| --- | --- | --- | --- | --- | --- | --- |
|  |  |  | 2.5 mg | 5 mg | 10 mg |  |
| FAS, n | | 24 | 23 | 23 | 25 | 24 |
| Ht, mean±SD (n) | |  |  |  |  |  |
|  | Baseline level (%) | 30.89±4.28 (24) | 30.02±4.55 (22) | 31.24±3.23 (23) | 30.82±4.99 (25) | 29.91±6.06 (24) |
|  | Rate of change at 12 weeks (%) | 11.91±25.52 (20) | 15.88±22.32 (22) | 16.83±11.4 (21) | 19.8±19.39 (25) | 26.01±26.49 (23) |
|  | p value for trend* | >0.025 | | | | — |
| MCV, mean±SD (n) | |  |  |  |  |  |
|  | Baseline level (fL) | 78.3±8.3 (24) | 76.1±9.1 (22) | 78.4±7.7 (23) | 75.3±8.7 (25) | 76.4±11.3 (24) |
|  | Rate of change at 12 weeks (%) | 3.91±10.89 (20) | 1.73±7.99 (22) | -0.04±6.45 (21) | 3.48±10.88 (25) | 3.47±10.86 (23) |
|  | p value for trend* | >0.025 | | | | — |
| Ferritin, mean±SD (n) | |  |  |  |  |  |
|  | Baseline level (ng/dL) | 7.346±6.352 (24) | 7.186±7.764 (22) | 11.237±25.765 (23) | 7.794±9.639 (25) | 8.823±12.328 (24) |
|  | Rate of change at 12 weeks (%) | 80.84±165.05 (20) | 554.27±1893.82 (22) | 227.14±673.13 (21) | 426.38±776.61 (25) | 841.05±2269.5 (23) |
|  | p value for trend* | >0.025 | | | | — |
| TIBC, mean±SD (n) | |  |  |  |  |  |
|  | Baseline level (µg/dL) | 420.3±38.7 (24) | 415.5±65.3 (22) | 409.4±46.8 (23) | 417.4±66.4 (25) | 415.8±65.7 (24) |
|  | Rate of change at 12 weeks (%) | -0.53±10.68 (20) | -1.76±10.07 (22) | -2.25±10.18 (21) | -7.49±9.01 (25) | -2.06±11.5 (23) |
|  | p value for trend* | >0.025 | | | | — |

*p for trend between placebo and UPA groups, FAS: full analysis set, Ht: hematocrit, MCV: mean corpuscular volume, TIBC: total iron binding capacity
