# Supplementary material for: Ulipristal acetate for Japanese women with symptomatic uterine fibroids: A double‐blind, randomized, phase II dose‐finding study
Source: Reprod Med Biol. 2019 Oct 30;19(1):65–74. doi: 10.1002/rmb2.12304 (PMC6955589; doi:10.1002/rmb2.12304)
Supplement: Supplementary file 3 [file RMB2-19-65-s003.docx]

Supporting Table 3. VAS for pain in full analysis set

|  | | Placebo | Ulipristal | | | Leuprorelin |
| --- | --- | --- | --- | --- | --- | --- |
|  |  |  | 2.5 mg | 5 mg | 10 mg |  |
| FAS (n) | | 24 | 22 | 23 | 25 | 24 |
| Amount change in VAS (mm), mean±SD (n) | |  |  |  |  |  |
|  | 4 weeks | 5.0±21.1 (24) | -6.7±18.7 (22) | -3.9±20.9 (23) | -10.3±26.5 (24) | -11.8±30.0 (24) |
|  | 8 weeks | 0.1±34.0 (23) | -4.7±11.6 (21) | -4.8±21.4 (23) | -11.4±25.5 (25) | -14.3±30.0 (24) |
|  | 12 weeks | 6.0±23.9 (22) | 2.4±17.4 (21) | -7.1±21.4 (22) | -7.8±30.9 (24) | -18.0±29.1 (23) |
|  | 24 weeks | -12.8±30.3 (5) | 4.2±39.4 (11) | -0.4±25.5 (12) | -14.0±25.1 (7) | -21.3±29.8 (6) |
|  | p value for trend* | 0.1004 | | | | — |

*p for trend between placebo and UPA groups, FAS: full analysis set, VAS: visual analogue scale
