# Supplementary material for: Ulipristal acetate for Japanese women with symptomatic uterine fibroids: A double‐blind, randomized, phase II dose‐finding study
Source: Reprod Med Biol. 2019 Oct 30;19(1):65–74. doi: 10.1002/rmb2.12304 (PMC6955589; doi:10.1002/rmb2.12304)
Supplement: Supplementary file 4 [file RMB2-19-65-s004.docx]

Supporting Table 4. Quality of life

|  | | Placebo | Ulipristal | | | Leuprorelin |
| --- | --- | --- | --- | --- | --- | --- |
|  |  |  | 2.5 mg | 5 mg | 10 mg |  |
| Physical component summary score, mean±SD (n) | |  |  |  |  |  |
|  | Baseline | 48.13±10.21 (24) | 46.62±9.33 (22) | 46.77±9.05 (23) | 46.18±9.46 (25) | 49.22±6.14 (24) |
|  | 4 weeks | 46.65±9.65 (24) | 48.88±9.15 (22) | 48.65±10.99 (23) | 50.03±6.66 (23) | 51±6.27 (24) |
|  | 12 weeks | 45.36±11.16 (22) | 48.27±7.93 (21) | 52.07±7.32 (22) | 50.4±10.42 (24) | 51.44±6.6 (23) |
|  | 24 weeks | 50.85±7.29 (5) | 48.39±11.61 (11) | 45.75±10.05 (12) | 48.63±10.02 (7) | 53.37±7.23 (6) |
|  | p value for trend* | >0.025 | | | | — |
| Mental component summary score, mean±SD (n) | |  |  |  |  |  |
|  | Baseline | 46.48±8.02 (24) | 48.33±7.43 (22) | 48.89±8.54 (23) | 48.86±9.45 (25) | 47.65±8.3 (24) |
|  | 4 weeks | 46.69±7.99 (24) | 50.43±10.05 (22) | 51.42±7.37 (23) | 50.46±8.53 (23) | 48.89±7.02 (24) |
|  | 12 weeks | 44.41±8.39 (22) | 52.6±7 (21) | 49.99±6.03 (22) | 49.61±8.25 (24) | 47.46±9.28 (23) |
|  | 24 weeks | 46.31±6.28 (5) | 50.41±7.12 (11) | 51.36±4.46 (12) | 54.39±6.89 (7) | 42.79±8.52 (6) |
|  | p value for trend* | >0.025 | | | | — |
| Role/Social component summary score, mean±SD (n) | |  |  |  |  |  |
|  | Baseline | 41.96±11.24 (24) | 47.43±9.77 (22) | 48.07±13.41 (23) | 50.32±8.89 (25) | 48.09±9.65 (24) |
|  | 4 weeks | 41.84±10.68 (24) | 45.28±11.99 (22) | 49.66±9.53 (23) | 50.61±7.76 (23) | 48.11±8.2 (24) |
|  | 12 weeks | 40.7±10.97 (22) | 49.41±7.33 (21) | 46.68±10.55 (22) | 49.87±8.89 (24) | 45.45±9.94 (23) |
|  | 24 weeks | 40.04±10.92 (5) | 51.09±7.25 (11) | 45.13±7.33 (12) | 53.03±6.98 (7) | 46.54±12.09 (6) |
|  | p value for trend* | >0.025 | | | | — |

*p for trend between placebo and UPA groups
