# Supplementary material for: Ulipristal acetate for Japanese women with symptomatic uterine fibroids: A double‐blind, randomized, phase II dose‐finding study
Source: Reprod Med Biol. 2019 Oct 30;19(1):65–74. doi: 10.1002/rmb2.12304 (PMC6955589; doi:10.1002/rmb2.12304)
Supplement: Supplementary file 5 [file RMB2-19-65-s005.docx]

Supporting Table 5. Endocrine tests in safety analysis set

|  | | Placebo | | Ulipristal | | | | | | Leuprorelin | |  |
| --- | --- | --- | --- | --- | --- | --- | --- | --- | --- | --- | --- | --- |
|  |  |  |  | 2.5 mg | | 5 mg | | 10 mg | |  |  |  |
| SAF （n） | | 24 | | 23 | | 23 | | 25 | | 24 | |  |
| ACTH (pg/mL), mean±SD (n) | |  | |  | |  | |  | |  | |  |
|  | Pre-treatment | | 22.16±12.51 (24) | | 20.09±11.52 (23) | | 18.77±9.94 (23) | | 17.74±8.06 (25) | | 20.99±10.58 (24) | |
|  | Baseline | | 16.63±11.52 (24) | | 18.81±8.34 (23) | | 18.24±9.23 (23) | | 17.94±6.97 (25) | | 19.32±8.05 (24) | |
|  | 4 weeks | | 17.65±7.65 (24) | | 16.59±9.56 (23) | | 17.77±7.85 (23) | | 16.36±7.23 (25) | | 19.22±7.68 (24) | |
|  | 8 weeks | | 18.6±9.56 (22) | | 17.2±7.03 (23) | | 19.15±8.51 (23) | | 17.94±9.03 (25) | | 19.08±7.19 (24) | |
|  | 12 weeks | | 16.53±6.7 (20) | | 18.39±10.18 (23) | | 18.74±8.65 (21) | | 15.68±8.49 (25) | | 18.12±7.13 (23) | |
|  | 16 weeks | | 16.4±8.13 (19) | | 15.07±8.59 (21) | | 21.77±10.21 (21) | | 17.55±9.18 (25) | | 22.83±13.74 (22) | |
|  | 24 weeks | | 20.63±13.66 (18) | | 19.28±6.88 (21) | | 18.28±9.52 (21) | | 19.3±10.52 (25) | | 20.03±12.53 (22) | |
| TSH (µIU/mL), mean±SD (n) | |  | |  | |  | |  | |  | |  |
|  | Pre-treatment | 1.7858±1.1913 (24) | | 1.9957±0.8996 (23) | | 1.7382±1.0416 (23) | | 1.5517±0.7644 (25) | | 1.7311±1.0045 (24) | |  |
|  | Baseline | 1.6139±0.6873 (24) | | 1.9158±1.0391 (23) | | 1.5963±1.0583 (23) | | 1.7121±0.8373 (25) | | 1.6495±0.9905 (24) | |  |
|  | 4 weeks | 1.5767±0.85 (24) | | 1.877±0.9098 (23) | | 1.4355±0.8607 (23) | | 1.7017±0.8531 (25) | | 1.7702±0.8659 (24) | |  |
|  | 8 weeks | 2.1187±1.7395 (22) | | 1.7626±0.901 (23) | | 1.4985±1.1976 (23) | | 1.7956±0.7233 (25) | | 2.1342±1.7901 (24) | |  |
|  | 12 weeks | 2.0654±1.3559 (20) | | 2.959±3.3888 (23) | | 1.3896±0.6949 (21) | | 1.895±0.8168 (25) | | 1.9901±1.5779 (23) | |  |
|  | 16 weeks | 1.7253±0.8826 (19) | | 3.1353±6.3193 (21) | | 1.6293±1.1478 (21) | | 1.9355±1.0592 (25) | | 2.1042±1.5243 (22) | |  |
|  | 24 weeks | 1.8481±1.1588 (18) | | 2.1472±1.795 (21) | | 1.7305±1.2419 (21) | | 1.6703±1.1648 (25) | | 1.8318±1.1323 (22) | |  |
| FSH (µIU/mL), mean±SD (n) | |  | |  | |  | |  | |  | |  |
|  | Pre-treatment | | 7.062±5.576 (24) | | 5.268±1.931 (23) | | 7.27±9.331 (23) | | 6.494±5.181 (25) | | 8.045±9.582 (24) | |
|  | Baseline | | 8.547±6.502 (24) | | 7.404±4.8 (23) | | 8.607±8.134 (23) | | 11.224±8.519 (25) | | 9.196±5.667 (24) | |
|  | 4 weeks | | 8.312±6.96 (24) | | 3.92±1.772 (23) | | 3.155±1.935 (23) | | 2.61±2.265 (25) | | 2.613±1.249 (24) | |
|  | 8 weeks | | 5.587±2.685 (22) | | 3.792±2.338 (23) | | 5.223±6.907 (23) | | 4.946±6.911 (25) | | 3.969±1.479 (24) | |
|  | 12 weeks | | 8.329±10.083 (20) | | 5.072±4.683 (23) | | 4.045±2.868 (21) | | 4.142±2.408 (25) | | 4.835±1.411 (23) | |
|  | 16 weeks | | 5.564±2.762 (19) | | 6.406±4.799 (21) | | 6.426±5.773 (21) | | 6.064±5.056 (25) | | 7.638±6.254 (22) | |
|  | 24 weeks | | 8.112±6.35 (18) | | 4.667±2.306 (21) | | 7.376±10.323 (21) | | 8.704±8.888 (25) | | 8.38±10.136 (22) | |
| LH (µIU/mL), mean±SD (n) | |  | |  | |  | |  | |  | |  |
|  | Pre-treatment | | 7.006±6.945 (24) | | 7.417±8.813 (23) | | 6.713±6.597 (23) | | 8.137±9.997 (25) | | 6.496±7.087 (24) | |
|  | Baseline | | 4.348±3.281 (24) | | 4.532±2.158 (23) | | 5.299±5.906 (23) | | 4.499±1.773 (25) | | 4.133±2.439 (24) | |
|  | 4 weeks | | 7.249±11.918 (24) | | 4.19±2.967 (23) | | 5.134±3.588 (23) | | 5.631±4.171 (25) | | 0.568±0.295 (24) | |
|  | 8 weeks | | 4.408±3.552 (22) | | 4.839±2.443 (23) | | 6.192±5.859 (23) | | 5.634±3.369 (25) | | 0.274±0.214 (24) | |
|  | 12 weeks | | 6.76±9.279 (20) | | 6.253±3.581 (23) | | 4.631±2.851 (21) | | 5.766±3.831 (25) | | 0.28±0.282 (23) | |
|  | 16 weeks | | 3.977±2.075 (19) | | 4.87±2.878 (21) | | 6.242±7.966 (21) | | 5.311±4.945 (25) | | 2.069±1.663 (22) | |
|  | 24 weeks | | 11.5±20.257 (18) | | 4.548±2.626 (21) | | 5.688±6.509 (21) | | 7.686±10.858 (25) | | 5.192±4.099 (22) | |
| Prolactin (ng/mL), mean±SD (n) | |  | |  | |  | |  | |  | |  |
|  | Pre-treatment | | 15.215±8.666 (24) | | 18.537±15.751 (23) | | 14.917±6.601 (23) | | 15.88±6.396 (25) | | 14.85±7.623 (24) | |
|  | Baseline | | 13.915±7.042 (24) | | 14.719±9.244 (23) | | 12.618±4.946 (23) | | 14.66±5.684 (25) | | 12.433±6.905 (24) | |
|  | 4 weeks | | 14.795±5.983 (24) | | 15.312±11.546 (23) | | 13.967±6.592 (23) | | 16.117±8.209 (25) | | 10.612±4.954 (24) | |
|  | 8 weeks | | 14.459±5.612 (22) | | 16.614±11.123 (23) | | 14.921±8.058 (23) | | 13.32±4.442 (25) | | 10.158±4.606 (24) | |
|  | 12 weeks | | 14.557±6.194 (20) | | 18.558±18.64 (23) | | 12.558±5.872 (21) | | 12.792±8.402 (25) | | 9.676±4.344 (23) | |
|  | 16 weeks | | 13.631±4.668 (19) | | 14.509±8.765 (21) | | 16.119±10.943 (21) | | 14.516±7.435 (25) | | 11.105±4.828 (22) | |
|  | 24 weeks | | 17.23±8.056 (18) | | 18.194±11.603 (21) | | 14.279±6.901 (21) | | 13.324±6.066 (25) | | 12.451±7.389 (22) | |

SAF: safety analysis set, ACTH: adrenocorticotropic hormone, TSH: thyrotropin-releasing hormone, FSH: follicle stimulating hormone, LH: luteinizing hormone
