# Supplementary material for: Ulipristal acetate for Japanese women with symptomatic uterine fibroids: A double‐blind, randomized, phase II dose‐finding study
Source: Reprod Med Biol. 2019 Oct 30;19(1):65–74. doi: 10.1002/rmb2.12304 (PMC6955589; doi:10.1002/rmb2.12304)
Supplement: Supplementary file 6 [file RMB2-19-65-s006.docx]

Supporting Table 6. Bone turnover markers in safety analysis set

|  | | Placebo | Ulipristal | | | Leuprorelin |
| --- | --- | --- | --- | --- | --- | --- |
|  |  |  | 2.5 mg | 5 mg | 10 mg |  |
| SAF （n） | | 24 | 23 | 23 | 25 | 24 |
| PINP (µg/L), mean±SD (n) | |  |  |  |  |  |
|  | Baseline | 44.95±14.85 (24) | 43.11±12.78 (23) | 50.18±21.11 (23) | 48.85±17.11 (25) | 40.76±14.96 (24) |
|  | 8 weeks | 54.71±19.93 (22) | 55.67±32.86 (23) | 71.32±65.86 (23) | 57.01±20.49 (25) | 34.2±11.01 (24) |
|  | 12 weeks | 52.18±17.29 (20) | 54.02±20.52 (23) | 58.05±28.83 (21) | 56.13±21.35 (25) | 44.26±14.6 (23) |
| BAP (µg/L), mean±SD (n) | |  |  |  |  |  |
|  | Baseline | 9.5±3.7 (24) | 8.83±2.33 (23) | 9.4±2.04 (23) | 9.42±3.67 (25) | 8.96±2.28 (24) |
|  | 8 weeks | 9.84±3.68 (22) | 9.02±2.11 (23) | 9.63±2.35 (23) | 10.5±4.04 (25) | 10.17±2.85 (24) |
|  | 12 weeks | 9.81±3.17 (20) | 9.16±1.87 (23) | 10.15±2.08 (21) | 10.22±3.48 (25) | 11.39±3.06 (23) |
| Deoxypiridinoline (nmol/mmol Cr), mean±SD (n) | |  |  |  |  |  |
|  | Baseline | 5.75±0.9 (24) | 5.27±1.43 (23) | 6.33±1.89 (23) | 6.14±1.71 (25) | 5.78±1.74 (24) |
|  | 8 weeks | 5.76±1.53 (22) | 6.68±2.02 (23) | 8.16±5.52 (23) | 7.23±2.02 (25) | 7.68±1.9 (24) |
|  | 12 weeks | 5.6±1.46 (20) | 6.19±2.6 (23) | 7.97±3.99 (21) | 7.44±2.22 (25) | 8.24±1.75 (23) |
| CTX (µg/mmol Cr), mean±SD (n) | |  |  |  |  |  |
|  | Baseline | 133.388±70.105 (24) | 116.278±54.667 (23) | 134.848±53.685 (23) | 118.48±66.394 (25) | 118.758±79.216 (24) |
|  | 8 weeks | 147.773±102.345 (22) | 122.183±55.797 (23) | 189.8±100.444 (23) | 174.632±95.332 (25) | 250.329±108.715 (24) |
|  | 12 weeks | 158.613±93.526 (20) | 130.109±74.143 (23) | 177±77.447 (21) | 174.468±80.601 (25) | 268.657±108.09 (23) |

SAF: safety analysis set, PINP: type I procollagen N-terminal propeptide, BAP: bone alkaline phosphatase, CTX: type I collagen C-terminal telopeptide
