# Supplementary material for: Ulipristal acetate for Japanese women with symptomatic uterine fibroids: A double‐blind, randomized, phase II dose‐finding study
Source: Reprod Med Biol. 2019 Oct 30;19(1):65–74. doi: 10.1002/rmb2.12304 (PMC6955589; doi:10.1002/rmb2.12304)
Supplement: Supplementary file 7 [file RMB2-19-65-s007.docx]

Supporting Table 7. Study organization

| Centralized Gynecological Assessment Committee | | |  |
| --- | --- | --- | --- |
|  | Hiroshi Fujiwara | Kanazawa University Hospital | |
|  | Tetsuo Maruyama | Keio University Hospital | |
|  | Koji Kugu | Tokyo Metropolitan Bokutoh Hospital | |
| Centralized Image Assessment Committee | | |  |
|  | Kaori Togashi | Kyoto University | |
|  | Aki Kido | Kyoto University Hospital | |
| Centralized Histopathological Assessment Committee | | |  |
|  | Yoshiki Mikami | Kumamoto University Hospital | |
|  | Takako Kiyokawa | The Jikei University | |
|  | Masanori Yasuda | Saitama Medical University International Medical Center | |
| Study sites and investigators | | |  |
|  | Teruko Yasuda | Yoshio Obstetrics Gynecology Hospital | |
|  | Masaki Hashimoto | Hashimoto Clinic | |
|  | Tomoe Tando | Hirosaki National Hospital | |
|  | Hideyoshi Matsumura | Shinshu Ueda Medical Center | |
|  | Motomu Ando | Kugayama hospital | |
|  | Naoaki Kuji | Tokyo Medical University Hospital | |
|  | Kenji Sato | Keio University Hospital | |
|  | Mineto Morita | Toho University Omori Medical Center | |
|  | JunKumakiri | Juntendo University Hospital | |
|  | Tomonori Ishikawa | Tokyo Medical And Dental University | |
|  | Yasushi Hirota | The University of Tokyo Hospital | |
|  | Shigeo Akira | Nippon Medical School Hospital | |
|  | Youichi Kobayashi | Kyorin University Hospital | |
|  | Sigeto Yamauchi | Ota General Hospital | |
|  | Yoshi Kubota | Yokohama Medical Center | |
|  | Kiyoshi Takamatsu | Tokyo Dental College Ichikawa General Hospital | |
|  | Kouyou Yoshida | Juntendo University Urayasu Hospital | |
|  | Hiroyuki Nakagawa | Saitama National Hospital | |
|  | Yasushi Takai | Saitama Medical Center | |
|  | Akira Iwase | Nagoya University Hospital | |
|  | Koichi Shinohara | Aichi Medical University Hospital | |
|  | Toshikazu Yoshimura | Yachiyo Hospital | |
|  | Yuji Suto | Sutou Ladies Clinic | |
|  | Hisato Oku | Chayamachi Ladies Clinic | |
|  | Kenjiro Sawada | Osaka University Hospital | |
|  | Yoshito Terai | Osaka Medical College Hospital | |
|  | Takashi Inoue | Higashi-Ohmi General Medical Center | |
|  | Eiji Kondo | Kyoto University Hospital | |
|  | Shunji Nojima | Kanazawa Medical Center | |
|  | Kyousuke Takeuchi | Kobe Medical Center | |
|  | Seiichi Nishikawa | Nishikawa Ladies Clinic | |
|  | Toshiya Matsuzaki | Tokushima University Hospital | |
|  | Ichiro Yasuhi | Nagasaki Medical Center | |
|  | Michio Kitajima | Nagasaki University Hospital | |
|  | Akira Fujishita | Saiseikai Nagasaki Hospital | |
|  | Ritsuo Honda | Kumamoto University Hospital | |
|  | Hiroshi Nishimura | Kumamoto Medical Center | |
|  | Hisao Sumioki | Beppu Medical Center | |

The affiliations of the investigators are at the time of the study
